# A forecasting model for dengue incidence in the District of Gampaha, Sri Lanka

**DOI:** 10.1186/s13071-018-2828-2

**Published:** 2018-04-24

**Authors:** Gayan P. Withanage, Sameera D. Viswakula, Y. I. Nilmini Silva Gunawardena, Menaka D. Hapugoda

**Affiliations:** 1 Molecular Medicine Unit, Faculty of Medicine, University of Kelaniya, Ragama, Sri Lanka; 2 Department of Statistics, Faculty of Science, University of Colombo, Colombo 03, Sri Lanka

**Keywords:** Dengue, District of Gampaha, Prediction model, Time series regression

## Abstract

**Background:**

Dengue is one of the major health problems in Sri Lanka causing an enormous social and economic burden to the country. An accurate early warning system can enhance the efficiency of preventive measures. The aim of the study was to develop and validate a simple accurate forecasting model for the District of Gampaha, Sri Lanka. Three time-series regression models were developed using monthly rainfall, rainy days, temperature, humidity, wind speed and retrospective dengue incidences over the period January 2012 to November 2015 for the District of Gampaha, Sri Lanka. Various lag times were analyzed to identify optimum forecasting periods including interactions of multiple lags. The models were validated using epidemiological data from December 2015 to November 2017. Prepared models were compared based on Akaike’s information criterion, Bayesian information criterion and residual analysis.

**Results:**

The selected model forecasted correctly with mean absolute errors of 0.07 and 0.22, and root mean squared errors of 0.09 and 0.28, for training and validation periods, respectively. There were no dengue epidemics observed in the district during the training period and nine outbreaks occurred during the forecasting period. The proposed model captured five outbreaks and correctly rejected 14 within the testing period of 24 months. The Pierce skill score of the model was 0.49, with a receiver operating characteristic of 86% and 92% sensitivity.

**Conclusions:**

The developed weather based forecasting model allows warnings of impending dengue outbreaks and epidemics in advance of one month with high accuracy. Depending upon climatic factors, the previous month’s dengue cases had a significant effect on the dengue incidences of the current month. The simple, precise and understandable forecasting model developed could be used to manage limited public health resources effectively for patient management, vector surveillance and intervention programmes in the district.

**Electronic supplementary material:**

The online version of this article (10.1186/s13071-018-2828-2) contains supplementary material, which is available to authorized users.

## Background

Dengue is the most rapidly spreading mosquito-borne viral infection in the world causing more than 390 million dengue infections annually of which 96 million clinically manifest. The disease mainly appears in tropical and subtropical regions of the world and approximately 3.9 billion people are living in these dengue endemic countries [1, 2]. The causative agent of the disease is one of the four serotypes of dengue virus (DENV) belonging to the genus *Flavivirus* of the family *Flaviviridae* and these viruses are transmitted to humans mainly *via* bites of female *Aedes* spp. mosquitoes, predominantly by *Ae. aegypti* (Linnaeus), while the subsidiary vector is *Ae. albopictus* (Skuse). The disease has a wide spectrum of clinical presentations, from undifferentiated dengue fever (DF), to dengue haemorrhagic fever (DHF), to life threatening dengue shock syndrome (DSS), creating significant health, economic and social burdens in endemic areas. Large-scale unplanned and uncontrolled urbanization, together with a rapid increase in the human population, leads to higher transmission of the disease in endemic areas [3–6].

In Sri Lanka, dengue is the most important vector-borne disease. The first serologically confirmed dengue case was reported in 1962, and the largest dengue epidemic was reported in 2009, with 35,008 reported cases and 346 deaths, with an incidence rate of 170 per 100,000 population [7, 8]. According to the Epidemiological Unit of Sri Lanka, more than 30,000 cases were reported every year since 2012, and approximately half of these cases were reported from the Western Province, including the Colombo, Gampaha and Kalutara districts. The second highest number of dengue cases was reported from the District of Gampaha, Sri Lanka since 2010. Currently, all four DENV serotypes are circulating in the district. An intervention study conducted previously demonstrated the importance of community mobilization and waste management in controlling dengue in the District of Gampaha [9]. Another study demonstrated the efficacy of space spraying and destroying larvae to control *Aedes* dengue vector mosquitoes in the district, and the importance of public participation [10]. Despite much effort put into disease prevention and control measures, more than 5000 dengue cases on average were reported annually in the district, and more than 31,000 cases were reported in the year 2017 alone. In the absence of an effective drug or vaccine specific to the dengue virus, controlling of vectors at the adult and immature stages through eliminating breeding sources is the best method to control the transmission of dengue in the district.

Recently, many health authorities directed their attention toward an early warning system to reduce the incidence rates of dengue and to allocate scarce public health resources for effective intervention programmes. Predictive risk maps and mathematical models provide an alternative way of assessing and quantifying the distribution of risk factors and assessing interventions to predict impending dengue epidemics. These maps are predominantly beneficial in settings which lack sufficient data on disease surveillance [11]. Geographical information system (GIS)-based approaches and spatial statistical analysis can be deployed for the development of predictive risk maps. Further, climatic variables, which can affect life-cycles, survival rates and biting rates of mosquitoes, as well as the incubation period of DENV, are extensively studied as potential predictors and early warning tools of dengue distribution [12–15]. Previous studies have indicated that rainfall, temperature, and humidity play a greater role in dengue transmission, but a more precise weather-based forecasting tool will be required to assist national health authorities to perform effective vector control programmes and disease management [16–19].

## Methods

Risk maps and predictive models are not available yet for the District of Gampaha to assess the disease transmission and there is an urgent requirement for an early warning system to predict imminent dengue epidemics in the district. Therefore, this study aimed to analyze spatial and seasonal distribution of dengue incidence, and to propose a simple and precise dengue early warning system based on local meteorological factors in the district, using the time series regression method. The outcome of the study will probably improve the effectiveness of dengue surveillance programmes, ultimately controlling impending dengue epidemics in the district.

### Study area

The District of Gampaha is located adjacent to the District of Colombo and expands over 1387 km^2^. It is the second most populated district in Sri Lanka. The elevation of the district ranges from sea level to 450 m. The district comprises of fifteen Medical Officer of Health (MOH) areas, sectioned into 106 Public Health Inspector (PHI) areas, 1177 Grama Niladhari (GN) divisions with 1784 villages. All MOH areas in the district were considered for the study. The estimated human population of the district in 2008 is around 2.2 million with a 1.02% annual population growth rate. However, the population density varies in different MOH areas, depending on the presence of major cities and industrial sites in the district [9, 20].

### Data collection

**Fig. 1:**
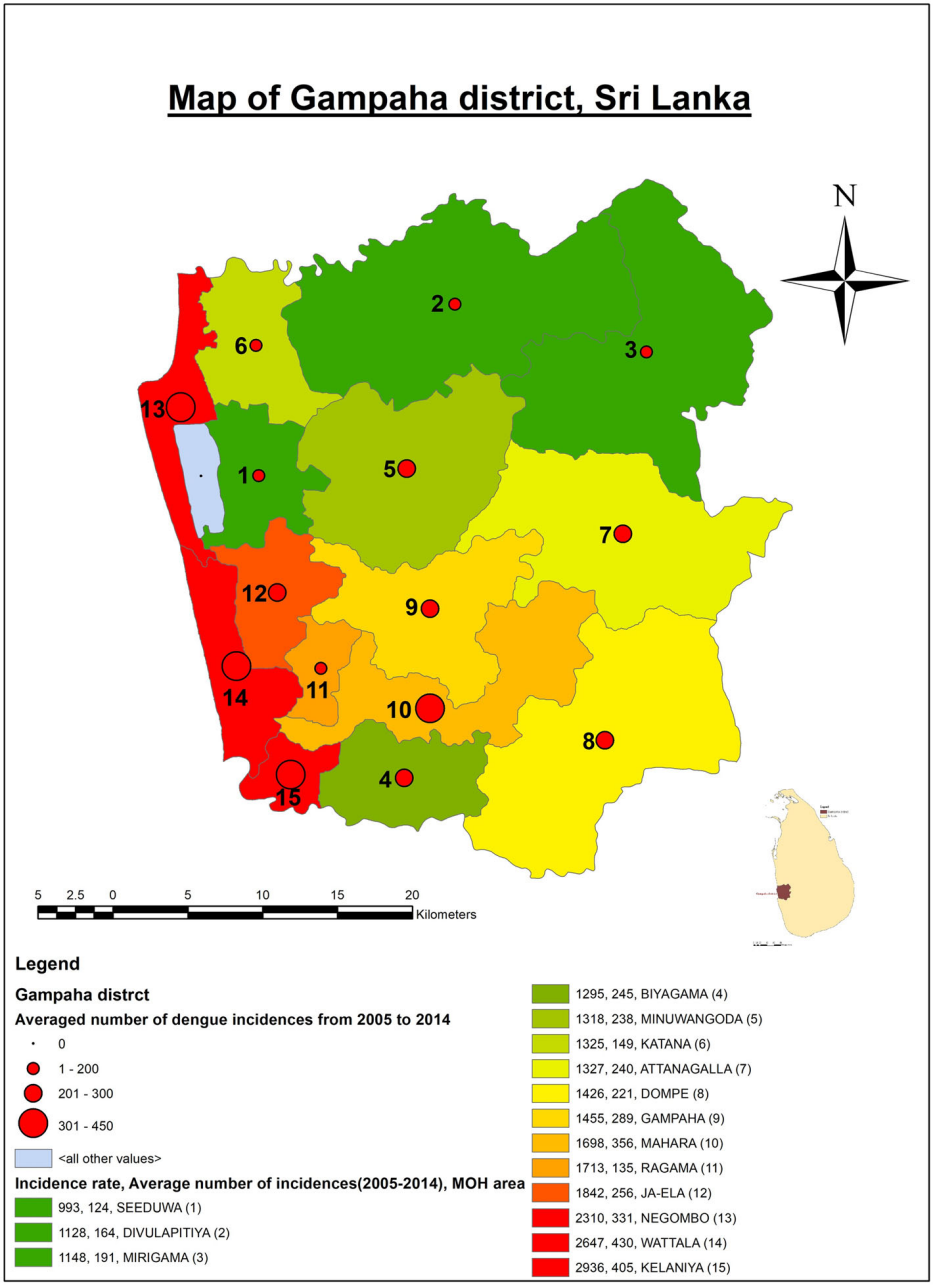
Incidence rate (cases per 100,000 population) and averaged number of dengue cases reported in District of Gampaha, Sri Lanka. Green color indicates the MOH areas with low dengue incidences rates, yellow indicates those with medium incidences rates, and red those with high incidences rates, from 2005 to 2014. The red colored dots indicate the average number of dengue cases reported in the each MOH area in this period

The reported number of dengue incidences was collected on monthly basis from the Regional Epidemiology Unit in the District of Gampaha for the period 2005 to 2014 in all fifteen MOH areas in the district. The total number of dengue cases was plotted yearly to identify patterns in the incidence distributions. Then, the average number of dengue cases was georeferenced in the district as claimed by each MOH area. Figure 1 shows the incidence rate per 100,000 population and the average number of dengue case reported from 2005 to 2014 in each MOH area in the District of Gampaha.

Weather records on rainfall, number of rainy days, minimum and maximum temperature, minimum and maximum Relative Humidity (RH), and wind speed were obtained from January 2012 to May 2017 from the Department of Meteorology, Colombo, Sri Lanka (where the climatic data are recorded centrally) on a monthly basis. These meteorological variables were collected by the weather stations at the Bandaranayake International Airport, Katunayake. In addition to that, rainfall and the number of rainy days were also collected from the Pasyala weather station. Rainfall data were highly correlated (*r*_*rainfall*_ = 0.77,  *r*_*rainy days*_ = 0.89) between the two weather stations, and the average values were taken for the analysis. Both weather stations are located in the middle of the district and they represent the study area well.

### Risk map construction

A risk map was developed based on the population density and average number of dengue incidences reported from 2005 to 2014 in each MOH area in the district, using ArcGIS software (v.10.2.1).

### Model construction

Multiple time series regression approaches were used to develop a dengue forecasting model. The data collected from January 2012 to November 2015 were used for the model construction and the observed data from December 2015 to May 2017 were used for validation of the models. Previous studies have mentioned relationships between monthly meteorological data and dengue incidence, from zero to three months lag periods considering the influence on mosquito survival and vertical disease transmission [21–23]. Therefore, several time series approaches were tried based on climatic variables with a lag of zero to three months and only three time series regression models are listed here. Time series regression models were fitted using R statistical software. The best model was selected based on lowest Akaike’s information criterion (AIC), Bayesian information criterion (BIC), R-squared (*R*^2^), root mean square errors (RMSE), mean absolute error (MAE), Pierce skill score (PSS) and mean absolute percentage error (MAPE) of prediction [24].

### Model 1

Here, a multiple time series regression model was fitted using log_10_ (cases) as the response variable and climatic variables with only the highest correlated lagged terms as the explanatory variables. Significant lagged terms of the climatic variables were found using the Pearson’s correlation test. Table 1 gives a summary of the correlation analysis.

**Table 1:** Pearson’s correlations between log_10_-transformed dengue cases (2012–2015) and weather variables in the District of Gampaha

Meteorological variable	Lag 0	Lag 1	Lag 2	Lag 3
Rainfall	-0.08	0.148	0.353*	0.094
Rainy days	-0.017	0.249	0.426*	0.065
Minimum temperature	0.066	0.25	0.291	0.295*
Maximum temperature	-0.385*	-0.398*	-0.238	0.058
Minimum RH	0.186	0.395*	0.420*	0.092
Maximum RH	-0.026	0.14	0.276	-0.019
Wind speed	0.159	-0.011	-0.234	-0.095
Dengue cases	–	0.716*	0.426*	0.222
(Dengue cases)^2^	–	0.720*	0.432*	0.226

*Significantly correlated lagged variables with dengue incidences during the study period. A 5% significance level was used to identify significant correlations

From Table 1, it can be seen that lag 2 of rainfall, lag 2 of rainy days, lag 3 of minimum temperature, lag 0 and 1 of maximum temperature, lag 1 and 2 of minimum RH, lag 1 and 2 of dengue cases, and lag 1 and 2 of squared dengue cases were significant at the 5% level. A multiple regression model was constructed using the stepwise method taking log_10_(*cases*)_*t*_ as the response variable and the highest correlated lagged variables listed in Table 1 as the explanatory variables. According to the stepwise method, the number of rainy days lagged two months (*Rainy days*_*t*−2_) and the squared number of dengue cases lagged one month (*cases*_*t*−1_^2^) showed a significant contribution to the distribution of dengue cases in the district. Therefore, our regression equation was:

1 $$ {y}_t={\beta}_0+{\beta}_1{x}_1{\left( Rainy\ days\right)}_{t-2}+{\beta}_2{x}_2{\left({(cases)}_{t-1}\right)}^2+{\varepsilon}_t\kern0.5em $$

where *y*_*t*_ is the dengue cases at time t, *β*_0_ is the baseline number of dengue cases derived from the multivariate model, *β*_1_ and *β*_2_are the regression coefficients for the rainy days lagged two months and the squared dengue cases lagged one month, respectively, and *ε*_*t*_ is the random error term of the model.

### Model 2

The dengue cases could be significantly associated with multiple lagged terms of retrospective meteorological variables and dengue incidences. The usual practice is to consider only the most significant lagged term and it may not be possible to capture the true variation of the response variable. Therefore, all climatic variables were considered up to three lags before proceeding to the model selection. Since the spread of recorded values was high in most variables, the explanatory variables were converted to log_10_ terms. Significant variables were identified using the stepwise regression method. In the selected model, significant climatic variables were rainfall, rainy days, and minimum temperature, all lagged 3 months, minimum RH lagged 1 and 2 months, and squared dengue cases lagged 1 month. Therefore, our regression equation for the model was:

2 $$ {y}_t={\beta}_0+{\beta}_1{x}_1{(Rainfall)}_{t-3}+{\beta}_2{x}_2{\left( Rainy\ days\right)}_{t-3}+{\beta}_3{x}_3{\left( Minimum\ temperature\right)}_{t-3}+{\beta}_4{x}_4{\left( Minimum\  RH\right)}_{t-2}+{\beta}_5{x}_5{\left( Minimum\  RH\right)}_{t-3}+{\beta}_6{x}_6{\left({(cases)}_{t-1}\right)}^2+{\varepsilon}_t\kern0.75em $$

where *y*_*t*_ is the dengue cases, *β*_0_ is the baseline number of dengue cases derived from the multivariate model, and *β*_1_to *β*_6_ are the regression coefficients for each respective variable. *ε*_*t*_ is the random error term in the regression model.

### Model 3

The third time series regression model was constructed using standardized meteorological data. Mean and standard deviation were calculated for each climatic variable from January 2012 to November 2015 and the climatic variables standardized. Then, stepwise regression model selection was performed with logarithm transformed dengue incidences *versus* retrospective standardized meteorological variables and logarithm transformed dengue incidences to identify significant variables. In the model, significant variables were rainfall and rainy days lagged 3 months, minimum RH lagged 2 months, and squared dengue cases lagged 1 month. Therefore, our regression equation for the model was:

3 $$ {y}_t={\beta}_0+{\beta}_1{x}_1{(Rainfall)}_{t-3}+{\beta}_2{x}_2{\left( Rainy\ days\right)}_{t-3}+{\beta}_3{x}_3{\left( Minimum\  RH\right)}_{t-2}+{\beta}_4{x}_4{\left({(cases)}_{t-1}\right)}^2+{\varepsilon}_t\kern6.75em $$

where *y*_*t*_ is the dengue cases, *β*_0_ is the baseline number of dengue cases derived from the multivariate model, and *β*_1_to *β*_4_ are the regression coefficients for each respective variable. *ε*_*t*_ is the error.

### Model validation

The augmented Dickey-Fuller (ADF) test was performed to identify the stationarity of and the presence of unit root of the residuals of the time series model. Autocorrelation function (ACF) and partial autocorrelation function (PACF) plots were analyzed for the residuals for each model. The good fit of the model was further examined by plotting the fitted values with reported dengue cases in the district.

### Forecasting of dengue case from the developed model

The developed models were used to forecast the dengue cases reported in the District of Gampaha from December 2015 to May 2017. Root mean squared error (RMSE), mean absolute error (MAE), mean absolute percentage difference (MAPE), and the Pierce skill score (PSS) (Additional file 1: Table S1) were calculated for the forecasts from each model and the forecasted values were plotted against reported dengue cases in the district during the forecasting period [25]. Sensitivity tests with receiver operating characteristics (ROC) were also performed for forecasting period to identify the sufficiency of the developed model [26–29].

## Results

### Overall dengue incidences

**Fig. 2:**
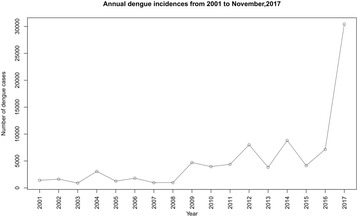
Annual number of dengue incidences from January 2001 to November 2017 for the District of Gampaha

There were 56,834 dengue incidences reported to the Epidemiology Unit, Sri Lanka, from all MOH areas in the District of Gampaha from January, 2001 to December, 2016. Figure 1 illustrates the population density and average number of dengue incidences in each MOH area reported from 2005 to 2014 in the district. The highest population density was observed from the Kelaniya MOH area along with more than 300 dengue incidences annually. Furthermore, high population densities were observed from Negombo, Wattala, Biyagama, and Ragama MOH areas. As shown in Fig. 2, more than half of the dengue incidences were reported recently during the period 2012–2016 even though there was a greater fluctuation. Also, the number of dengue incidences has increased significantly after 2012 compared to the period 2001–2011 (95% CI, *df* = 14, *P* = 0.001) and the study was, therefore, based on the dengue incidences during the period 2012–2016.

**Fig. 3:**
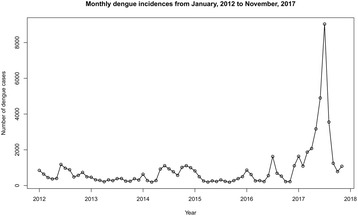
Monthly reported dengue incidences from January 2012 to November 2017 for the District of Gampaha

During the study period, 34,652 dengue incidences were recorded in the district with most incidences (31,637) in 2017. Figure 3 illustrates the monthly distribution of dengue incidences from January 2012 to May 2017 in the district. The distribution of dengue incidences was stationary according to the ADF test (*P* = 0.99).

### Forecasting dengue cases from December 2015 to November 2017

**Fig. 4:**
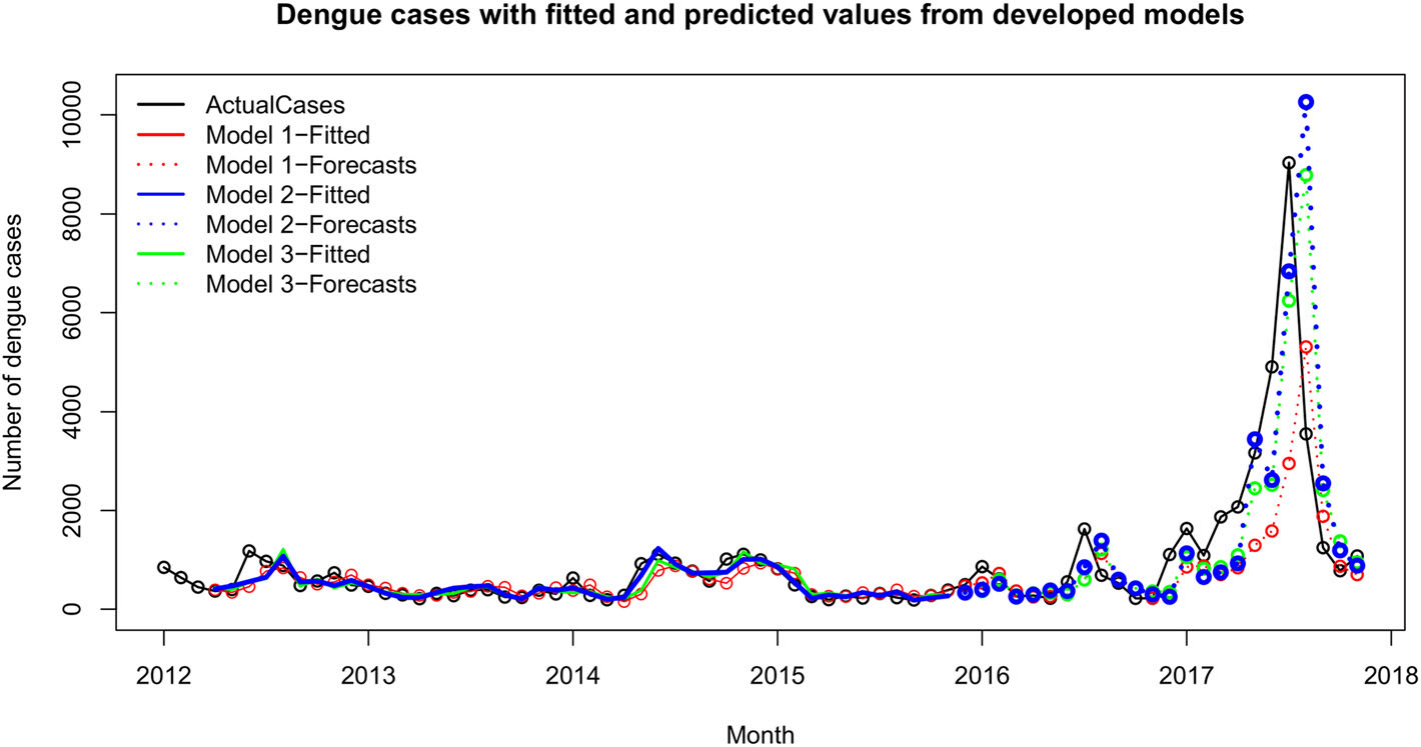
Fitted and forecast dengue cases from each developed model. Three models were developed and the fitted and forecast values from each model were plotted against actual dengue incidences. Actual incidences are shown in black, fitted values in each model are illustrated in different colored lines, and forecasts are shown in dotted line with respective color for each model

**Fig. 5:**
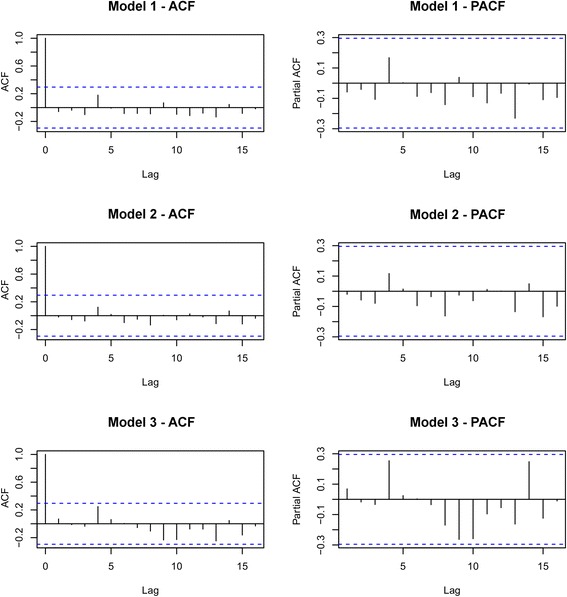
ACF and PACF of residuals of the developed forecasting models. ACF and PACF plots are shown on the left and right, respectively. There are no significant lags observed in ACF and PACF plots in the all three models

Figure 4 illustrates the distribution of dengue cases from each studied model with fitted cases and forecast dengue cases. The results of each model were summarized in Table 2. No significant lines were observed in the ACF and PACF plots in all three models and therefore, it was concluded that the residuals were distributed normally (Fig. 5).

**Table 2:** Results of the three models developed

Model	AIC	BIC	Adj. *R*^2^ (training)	Adj. *R*^2^ (testing)	For fitted (training) values			For forecasted (testing) values		
					MAE	RMSE	MAPE	MAE	RMSE	MAPE
Model 1	-33.59	-30.02	0.5611	0.4345	131.65	203.34	26.58	635.84	963.52	39.37
Model 2	-65.31	-61.74	0.7635	0.6880	95.65	146.83	18.81	532.39	715.59	43.76
Model 3	-50.01	-46.45	0.6824	0.7409	113.10	179.79	21.37	492.68	652.13	42.66

*Abbreviations*: *AIC* Akaike’s information criterion, *BIC* Bayesian information criterion, *Adj. R*^*2*^ adjusted *R*-square, *MAE* mean absolute error, *RMSE* root mean square error, *MAPE* mean absolute percentage error

According to Table 3, the lowest AIC and BIC and the highest *R*^2^ were achieved by Model 2. Therefore Model 2 was selected as the better model for the forecasting of dengue incidences in the District of Gampaha. According to Halide & Ridd [29], a moderate epidemic is defined when the number of dengue cases in a given month exceeds the 75th percentile. When the number of dengue cases exceeds the 90th percentile, it is defined as a severe epidemic. Based on the Gampaha district monthly data, the 75th and 90th percentiles were 977 and 1588, respectively. Therefore, in line with frequently used guidelines, 1200 was considered as the cutoff value for an outbreak [30]. The PSS was computed taking 1200 dengue cases as the cutoff for an epidemic. The PSS for the forecasts from Model 2 was 0.49, which indicates a moderate prediction ability of the model [31]. Parameter estimates of Model 2 are given in Table 3.

**Table 3:** Selected variables in the developed model, coefficients, standard errors and *P*-values

Variable	Coefficient	Standard error	*P*-value*
Intercept	-2.9881	2.11106	0.165298
Rainfall _t-3_	0.35815	0.10600	0.001728
Rainy days _t-3_	-0.66158	0.17185	0.000453
Minimum temperature _t-3_	3.3334	1.44787	0.027048
Minimum RH _t-2_	3.2757	1.05164	0.003546
Minimum RH _t-3_	-3.3490	1.24795	0.010828
(Dengue cases _t-1_)^2^	0.14715	0.01558	2.13E-11

*At 5% significance level

A regression equation was employed with the coefficients in Table 2 for the forecasting of dengue incidences with the developed model. The developed model was:

4 $$ \widehat{y}=-2.9881+0.35815{x}_1{(Rainfall)}_{t-3}-0.66158{x}_2{\left( Rainy\ days\right)}_{t-3}+3.3334{x}_3{\left( Minimum\ temperature\right)}_{t-3}+3.2757{x}_4{\left( Minimum\  RH\right)}_{t-2}-3.3490{x}_5{\left( Minimum\  RH\right)}_{t-3}+0.14715{x}_6{\left({(cases)}_{t-1}\right)}^2\kern4em $$

where $\widehat{y}$is the number of predicted dengue case. The forecast monthly dengue cases were plotted against the real-time reported cases in the district (Fig. 4).

**Fig. 6:**
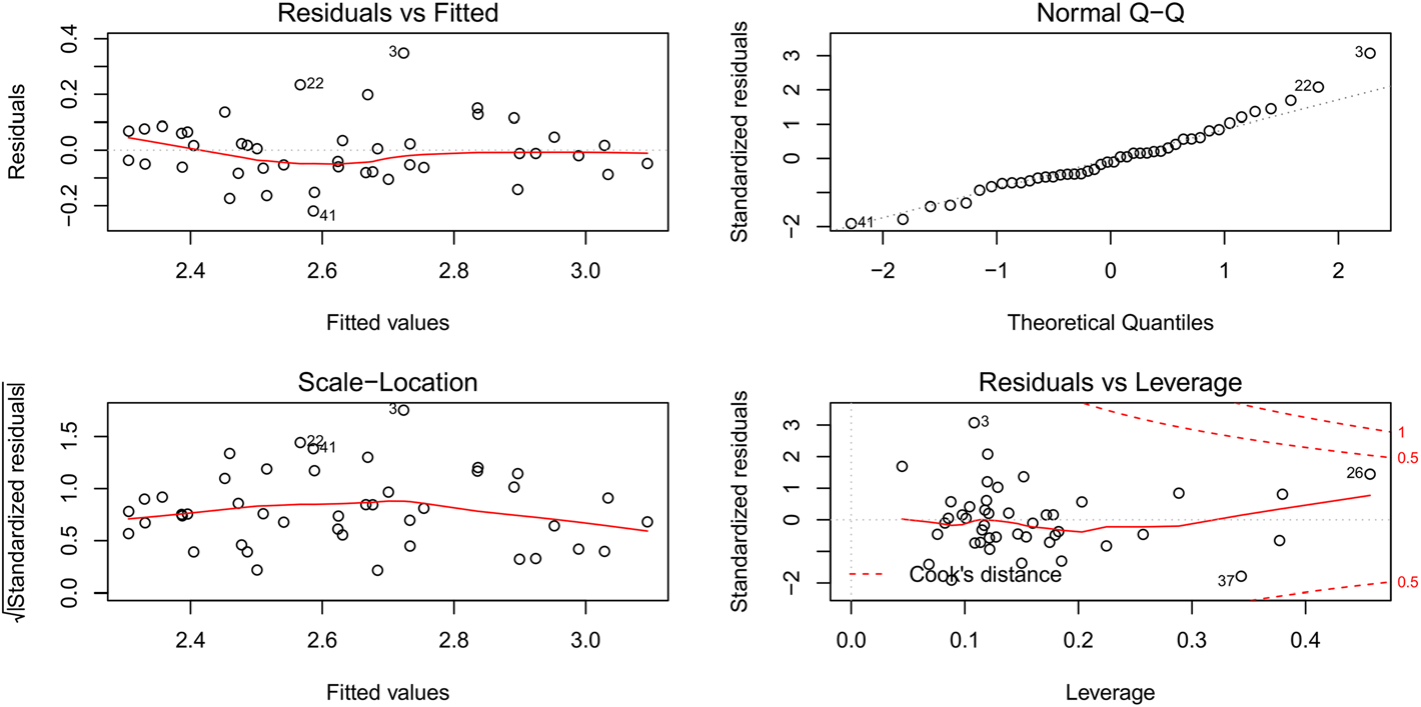
Residual normality plots of the developed model (Model 2). Approximately linear distributions of residuals are visualized in all plots. Errors were distributed around zero within ± 1 in the residual *vs* fitted plot

The distribution of residuals of the developed models was also analyzed (Fig. 6). Significant seasonality and trend could not be observed in the distribution of dengue cases. A realistically straight line can be observed in the residual normal probability plot, and the residual sequence plot illustrates a consistent distribution of errors around zero within ± 1. Furthermore, a symmetrical pattern can be observed in the residual histogram (Additional file 2: Figure S1). These observations indicate a normal distribution of residuals. There were no dengue epidemics observed in the district during the training period of the models, and nine outbreaks occurred during the forecasting period. The proposed model captured five outbreaks and rejected 14 of them correctly within the testing period of 24 months. The Pierce skill score of the model was 0.49, with a receiver operating characteristic of 86%, and 92% sensitivity.

## Discussion

Dengue incidences have been reported from every MOH area in the district. Adjacent MOH areas demonstrates similar disease transmission patterns analogous to their spatial and climatic similarity. Higher dengue incidence rates can be detected from highly congested MOH areas with the exception of the Ragama MOH area, which urbanized recently and has well-equipped health care facilities. The district experienced ample rainfall, fewer variations in temperature, and high humidity throughout the year, as it is located in the wet zone of a tropical country, which enhances the survival rates of *Aedes* mosquitoes. Therefore, meteorological variables were incorporated into the model as predictive variables. During the analysis of correlations between dengue incidences and climatic variables, monthly rainfall, number of rainy days, minimum and maximum RH, and minimum temperature show positive correlations while maximum temperature and wind speed show negative correlations.

The forecasting model developed in this study was based on retrospective monthly rainfall, rainy days, minimum temperature, minimum RH and dengue incidences. Even though monthly rainfall and rainy days are positively associated with the dengue cases, excessive and continuous rainfall may rinse out dengue vector mosquito breeding places and may delay the buildup of mosquito population until later in the rainy season, leading to a decrease in dengue incidences. A higher number of rainy days increased the accessible breeding places for vector mosquitoes leading to greater transmission of the disease [22, 32, 33]. Furthermore, a three-month lag time can increase the dengue vector mosquito populations as it allows sufficient time to complete the mosquito life-cycle, and acquire and proliferate the DENV in vector mosquitoes. These higher lag periods can partly be explained by survival of *Aedes* mosquito eggs in dry containers for several months and long egg hatching periods. In Pearson’s correlation analysis, a significant negative correlation was observed between dengue incidences with one month lagged maximum temperature. This also agreed with previous studies in Sri Lanka and Taiwan [8, 34]. The average maximum temperature in the district during the study period was 31.4 °C with a maximum of 34.3 °C. The negative correlation may be due to alteration of mosquito development, blood-feeding behavior, and availability of breeding containers [34]. Furthermore, a non-significant positive correlation was observed between dengue cases and minimum temperature. During the study period, the average minimum temperature was 24.5 °C which is favorable for mosquito survival and reproduction. The district experienced only minor fluctuations in temperature throughout the year. Additionally, a previous study in Sri Lanka reported that there was no correlation between small scale immediate temperature changes and impending dengue outbreaks [35]. However, the minimum temperature was significant at three months lag time in the selected model. This suggests the importance of carrying out a time series regression analysis considering serial correlations rather than shortsighted individual correlations obtained by a Pearson’s correlation analysis. Relative humidity is often associated with temperature and higher relative humidity increases mosquito population while lower relative humidity (e.g. 60%), will decrease the oviposition [36]. Both minimum and maximum relative humidity are higher in the district due to its close proximity to the Indian Ocean. Therefore, relative humidity is positively correlated with the transmission of dengue in the district as it increases the survival and oviposition rates in dengue vector mosquitoes [37]. Gampaha district frequently experiences light or gentle breezes, with wind speed two or three on the Beaufort wind scale. Wind speed is negatively correlated with dengue transmission as wind restricts flying activities of dengue vector mosquitoes, which affects human-mosquito contact and oviposition [38].

The current study has a few limitations. The District of Gampaha is located next to the District of Colombo, the capital of Sri Lanka, where the highest number of dengue cases is reported every year. Previous studies mentioned that during the daily commute of residents in the District of Gampaha to the District of Colombo for work and other commercial purposes, they can be infected by dengue. However, these patients are reported under the District of Gampaha [20]. There is no proper mechanism to track these commute related infections. Therefore, further studies are needed to focus on daily and monthly commutes of the district.

The district of Gampaha is located in the wet zone of the country with annual rainfall of 1400–2500 mm. The district experiences virtually the same precipitation throughout the year from the south-west and north-east monsoons, with a constant average temperature of 27.7 °C. The eastern part of the district is a hilly area with an elevation of about 150 m, while to the west, the elevation decreases steadily with lagoons, marsh lands to coastal areas. The district consists of five agro-ecological regions, wet zone low country WL1, WL2, WL3, WL4 and Intermediate zone low country IL1 [39]. The rainfall pattern differs in those regions, with highest expectancy of precipitation in WL1 and the lowest in IL1. Because of this, even though the number of dengue cases was reported by MOH areas, separate models need to be developed with significant variables particular to each MOH area to forecast impending dengue outbreaks in such areas.

Previous studies mentioned the importance of dengue incidences lagged one month to forecast current month dengue incidences [40–42]. Our model also illustrated that, depending upon climatic factors, the current month’s dengue cases were significantly affected by the previous month’s dengue cases in the district. Furthermore, there was a quadratic influence of the previous month’s dengue incidences on the current month’s dengue incidences in the District of Gampaha. This could also be a limitation of the selected model (Model 2). A further investigation was conducted to identify the effects of the previous month’s dengue incidences alone in forecasting the current month’s dengue incidences. In this model, the *R*^2^ for the training period was only 40.8% and the AIC and BIC values were higher compared to Model 2. Also, the forecast skill measured using PSS was 0.5. The model considering only the significant climatic variables (without lagged cases) resulted in an *R*^2^ of 19.3% for the training data. Additionally, higher AIC and BIC values suggested a lack of fit for the data (Additional file 1: Table S2). Therefore, it was confirmed that the selected model with lagged cases and significant explanatory variables was the better model for this study.

In this study, the effects of climatic factors and retrospective dengue incidences were modelled to forecast impending dengue outbreaks. However, dengue is a result of complex interactions of vectors, pathogens and humans. This includes introduction of new dengue viral genotypes, adaptations of dengue vector mosquitoes, commutes of population, herd immunity of population, urbanization, vegetation cover, land use patterns, efficiency of preventive and awareness programmes, socioeconomic factors and the attitude of people. The contribution of these factors to the magnitude of the dengue spread in the district still needs to be addressed. Moreover, there is insufficient time series data available to analyze the effects of these factors. Therefore, further investigations need to be conducted regarding these factors in a time series manner in the District of Gampaha.

## Conclusions

The model provides a one month time period for local vector surveillance and control agents, sufficient time to prepare for an impending dengue epidemic in the district in many ways. These early warning systems increase the efforts of dengue control during outbreaks reducing the impact of outbreak, disease transmission, healthcare burdens and possible mortalities. These simple, precise, and low-cost models can be utilized by end users, with more confidence in the early warning tool and minimum decision making time, allowing vector control units to utilize scarce public health resources for effective interventions. The higher precision of the model minimizes the use of health units on false dengue alarms, avoiding costly and unnecessary vector control operations. Dengue distribution patterns depend on many factors, such as ecological, environmental, epidemiological and social factors, and when a new vector control strategy and policies are implemented, these distribution patterns and epidemic cycles change over time. Therefore, appropriate modifications need to be made with re-calibration of the model according to the changes of risk factors and related fields, for long-term forecasting in future. Further studies need to be focused on the long-term sustainability of forecast precision and incorporation of the dengue early warning tool into the national dengue control system.

## Additional files

Additional file 1: **Table S1.** Contingency table used for to calculate the Pierce skill score. **Table S2.** Summary of the selected model with meteorological variables and without meteorological variables. (DOCX 20 kb)

Additional file 2: **Figure S1.** Histogram of residuals of Model 2. The approximate bell shape of the histogram indicates the normal distribution of residuals of the model. (PDF 4 kb)

## Abbreviations

ACF: autocorrelation function
ADF: augmented Dickey-Fuller test
AIC: Akaike’s information criterion
BIC: Bayesian information criterion
DENV: dengue virus
DF: dengue fever
DHF: dengue haemorrhagic fever
DSS: dengue shock syndrome
GIS: geographical information system
GN: Grama Niladhari
IL: intermediate zone low country
Log_10_: logarithm at base 10
MAE: mean absolute error
MAPE: mean absolute percentage error
MOH: Medical Officer of Health
PACF: partial autocorrelation function
PHI: Public Health Inspector
PSS: Pierce skill score
*R*^2^: R-squared
RH: relative humidity
RMSE: root mean square errors
ROC: receiver operating characteristics
WL: wet zone low country
