# Supplementary material for: A forecasting model for dengue incidence in the District of Gampaha, Sri Lanka
Source: Parasit Vectors. 2018 Apr 24;11:262. doi: 10.1186/s13071-018-2828-2 (PMC5916713; doi:10.1186/s13071-018-2828-2)
Supplement: Supplementary file 1 — Table S1. Contingency table used for to calculate the Pierce skill score. Table S2. Summary of the selected model with meteorological variables and without meteorological variables. (DOCX 20 kb) [file 13071_2018_2828_MOESM1_ESM.docx]

**Additional file 1**

**Pierce Skill Score (PSS)**

The Pierce skill score is a categorical type based skill measure of a developed model which consider both error estimates and random forecast score. It utilizes a Yes/No contingency tales as displayed in Table A1 [[25](#_ENREF_25), [29](#_ENREF_29), [31](#_ENREF_31)].

**A****dditional file 1: Table S1. Contingency table used for to calculate the Pierce skill score**

|  | | Observed | |
| --- | --- | --- | --- |
|  |  | Yes | No |
| Forecasted | Yes | *a* (correct forecasts) | *b* (false alarms) |
|  | No | *c* (misses) | *d* (correct negatives) |

Pierce skill score PSS = *(ad – bc) / (a + c) (b + d)*, where the total number of predictions and observations n = a + b + c + d [[29](#_ENREF_29)]. The skill score ranges from -1 to +1. A model will be useful if its prediction skill above 0.5.

**Comparison of the selected model with meteorological variables and without climatic variables**

**Additional file 1: Table S2. Summary of the selected model with meteorological variables and without meteorological variables**

| **Model** | **AIC** | **BIC** | **Adjusted R^2^**  **(training)** | **Adjusted R^2^**  **(testing)** | **For fitted (training) values** | | | | **For forecasted (testing) values** | | |
| --- | --- | --- | --- | --- | --- | --- | --- | --- | --- | --- | --- |
|  |  |  |  |  | **MAE** | **RMSE** | **MAPE** | **MAE** | | **RMSE** | **MAPE** |
| Model 2  (Selected Model) | -65.31 | -61.74 | 0.7635 | 0.6880 | 95.65 | 146.83 | 18.81 | 532.39 | | 715.59 | 43.76 |
| Model 2  Without climatic variables  (lag 1 cases Only) | -24.9 | -19.55 | 0.5012 | 0.4085 | 149.02 | 215.89 | 31.28 | 666.53 | | 985.36 | 46.83 |
| Model 2  Without lagged_1 dengue incidences  (Climatic variables Only) | -37.71 | -16.3 | 0.193 | -0.5112 | 202.17 | 265.71 | 41.71 | 1010.42 | | 1575.0 | 56.17 |
