# Supplementary figures and images for: A forecasting model for dengue incidence in the District of Gampaha, Sri Lanka
Source: Parasit Vectors. 2018 Apr 24;11:262. doi: 10.1186/s13071-018-2828-2 (PMC5916713; doi:10.1186/s13071-018-2828-2)

**Histogram of M2.1\$residuals**

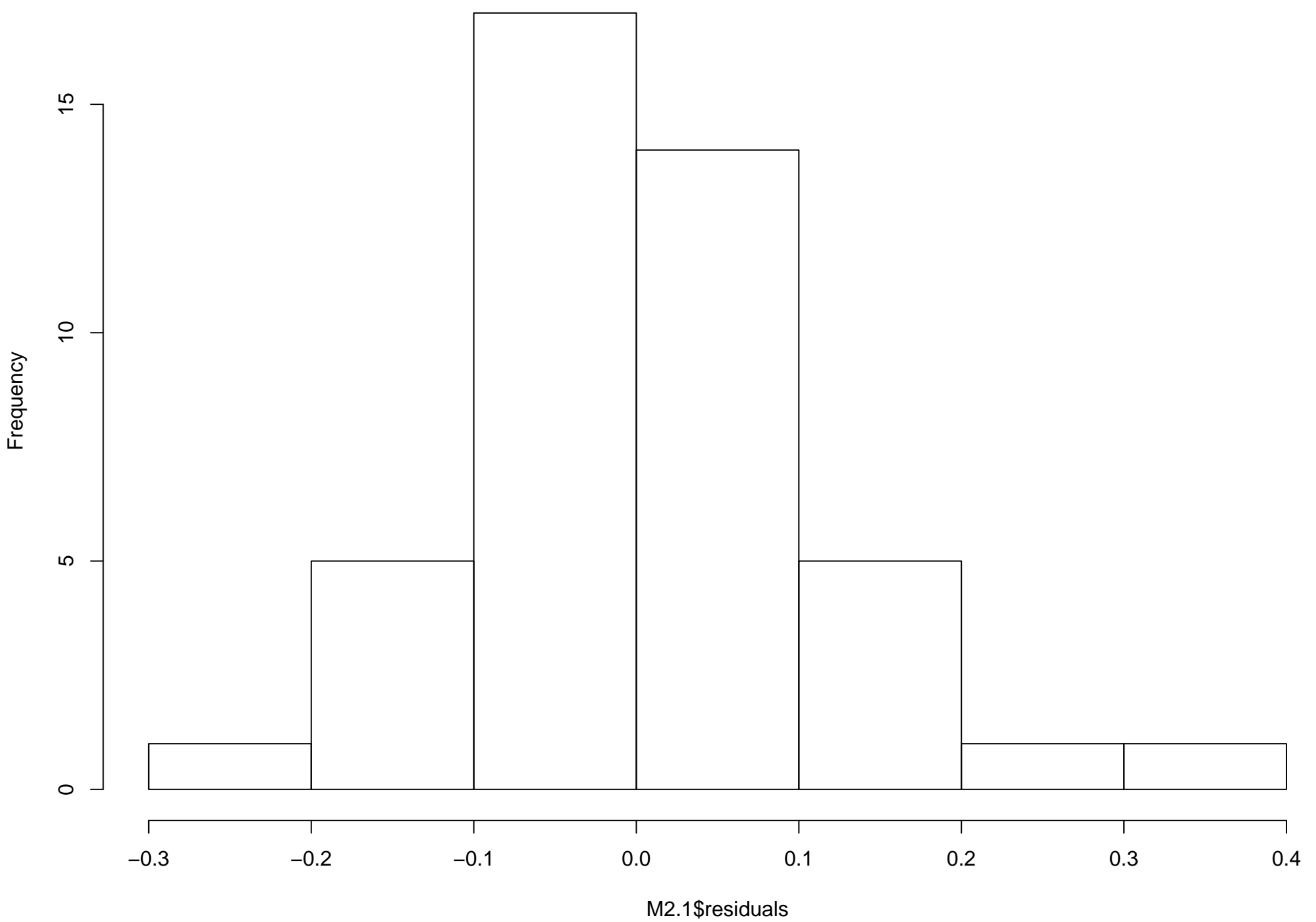

Supplement: Supplementary file 2 — Figure S1. Histogram of residuals of Model 2. The approximate bell shape of the histogram indicates the normal distribution of residuals of the model. (PDF 4 kb) [file 13071_2018_2828_MOESM2_ESM.pdf]
